# The Effects of Single Nucleotide Polymorphisms in Cancer RNAi Therapies

**DOI:** 10.3390/cancers12113119

**Published:** 2020-10-25

**Authors:** Magdalena Gebert, Maciej Jaśkiewicz, Adrianna Moszyńska, James F. Collawn, Rafał Bartoszewski

**Affiliations:** 1 Department of Biology and Pharmaceutical Botany, Medical University of Gdańsk, 80-416 Gdańsk, Poland; magdalena.gebert@gumed.edu.pl (M.G.); maciej.jaskiewicz@gumed.edu.pl (M.J.); adrianna.moszynska@gumed.edu.pl (A.M.); 2 Department of Cell, Developmental and Integrative Biology, University of Alabama at Birmingham, Birmingham, AL 35294, USA; jcollawn@uab.edu

**Keywords:** SNPs, RNAi therapy, gene therapy, siRNA, microRNA

## Abstract

**Simple Summary:**

Despite the recent progress in RNAi delivery of siRNA-based therapeutics for cancer therapy, the presence of single nucleotide polymorphisms (SNPs) in the general population could dramatically reduce the effectiveness of RNAi therapy. Their ubiquitous presence can also lead to unpredictable and adverse side effects. Because both SNPs and somatic mosaicisms have also been implicated in a number of human diseases including cancer, however, these specific changes offer the ability to selectively and efficiently target cancer cells. Here, we discuss how SNPs influence the development and success of novel anticancer RNAi therapies.

**Abstract:**

Tremendous progress in RNAi delivery methods and design has allowed for the effective development of siRNA-based therapeutics that are currently under clinical investigation for various cancer treatments. This approach has the potential to revolutionize cancer therapy by providing the ability to specifically downregulate or upregulate the mRNA of any protein of interest. This exquisite specificity, unfortunately, also has a downside. Genetic variations in the human population are common because of the presence of single nucleotide polymorphisms (SNPs). SNPs lead to synonymous and non-synonymous changes and they occur once in every 300 base pairs in both coding and non-coding regions in the human genome. Much less common are the somatic mosaicism variations associated with genetically distinct populations of cells within an individual that is derived from postzygotic mutations. These heterogeneities in the population can affect the RNAi’s efficacy or more problematically, which can lead to unpredictable and sometimes adverse side effects. From a more positive viewpoint, both SNPs and somatic mosaicisms have also been implicated in human diseases, including cancer, and these specific changes could offer the ability to effectively and, more importantly, selectively target the cancer cells. In this review, we discuss how SNPs in the human population can influence the development and success of novel anticancer RNAi therapies and the importance of why SNPs should be carefully considered.

## 1. Introduction

Cancer is a heterogenic group of diseases which occur through multistep alterations of oncogenes, tumor-suppressor genes, and microRNA genes and results in a disturbance of cellular homeostasis and changes cell differentiation and growth [1]. Notably, however, only few cancer-related proteins are accessible to modulation through monoclonal antibody-based drugs or small chemical molecules [2,3,4,5,6]. Furthermore, chemical targeting of cancer-related proteins is often accompanied by adverse side effects [7]. The discovery of RNA interference (RNAi) as a pathway for modulating mRNA stability provided a new opportunity to overcome the previous therapeutic limitations. Its importance was highlighted by the award of the Nobel Prize in Physiology and Medicine in 2006 to Andrew Z. Fire and Craig C. Mello [8]. It soon became clear that utilizing RNAi approaches allows for both rational drug design and control over chemically “undruggable” proteins that often contribute to human diseases. This led to the concept that RNAi drugs could be also used for interrupting carcinogenesis by reversing the cancer cell’s altered protein profiles through regulation of mRNA levels [9].

The initial clinical trials of RNAi drug candidates were unsuccessful because of the limitations of their immune-related toxicities, insufficient therapeutic efficacy, poor metabolic stability, and, perhaps most importantly, their off-target effects [10,11,12,13,14,15]. A critical turning point for the RNAi therapy, however, came with the introduction of the first approved small interfering RNA (siRNA) therapeutic, Onpattro (Patisiran, Alnylam Pharmaceuticals Inc). Patisiran uses a specific siRNA against transthyretin mRNA, and has been shown to reduce transthyretin protein levels sufficiently enough for the successful treatment of transthyretin-mediated amyloidosis [16,17]. Furthermore, two more drugs were approved. One was the RNAi-based Givosiran (Givlaari, Alnylam Pharmaceuticals Inc), an siRNA that targets 5-aminolevulinic acid synthase (enzyme crucial for the heme synthesis) mRNA [18,19] that is used for treatment of acute hepatic porphyria. The other was Golodirsen (Vyondys 53, Sarepta Therapeutics), an antisense phosphorodiamidate morpholino oligomer that is directed against a mutation in dystrophin pre-mRNA that restores the mRNA reading frame in patients with Duchenne muscular dystrophy [20].

Tremendous progress in RNAi delivery methods and design pipelines [4,21] include the evolution of siRNA-design dedicated software (reviewed in [22]), and this has allowed for the development of siRNA-based therapeutics that are currently under clinical investigation for cancer treatment (Table 1).

The use of RNAi drugs has the potential to revolutionize cancer pharmacotherapy by providing a unique ability to target the molecular pathomechanisms specific for individual patient’s cancer cells. This has the potential to provide efficient personalized therapy that is accompanied by minimal side effects compared to traditional chemotherapy.

One of the biggest advantages of RNAi drug candidates is their sequence specificity against the target genes. This exquisite specificity, however, also has a disadvantage. Genetic variations in the human population are common because of the presence of single nucleotide polymorphisms (SNPs) [23,24,25,26]. SNPs lead to synonymous and non-synonymous changes and they occur once in every 300 base pairs (bp) in both coding and non-coding regions in the human genome [27,28]. These changes, unfortunately, affect the RNAi interactions with their target mRNAs. Furthermore, the common phenomenon of the occurrence of genetically distinct populations of cells within an individual that is derived from postzygotic mutations called somatic mosaicism [21]. This heterogeneity may also affect the RNAi’s efficacy or more problematically, lead to unpredictable adverse side effects. On the other hand, both SNPs and somatic mosaicisms have been implicated in human diseases, including cancer [29], and this could offer the ability to specifically target the cancer cells. The overall goal of this article is to discuss how these mechanisms involving genetic variations in the human populations could either inhibit or enhance the development of novel anticancer RNAi therapies and to propose that SNPs should carefully be considered during these types of therapies.

## 2. RNA Interference

To fully appreciate the therapeutic potential of RNAi drugs, their mechanism of action needs to be discussed. RNAi is a native gene regulatory process in a majority of eukaryotic cells. Small non-coding RNA molecules (of different origin) are internally utilized for the management of gene expression or translation [34]. The short guide strands of ncRNAs can bind specific mRNA targets of homologous sequence and lead to mRNA post-transcriptional repression [35,36,37]. Notably, only 2% of the mammalian genome is transcribed into mRNA, whereas the majority of the genome is transcribed into ncRNAs [38,39], some of which can participate in RNAi. Based on the type of the ncRNAs’ guide strands and their biogenesis and mechanisms action on mRNA targets, three different categories of RNAi have been identified. These include double stranded RNAs (dsRNAs) such as the small interfering RNAs (siRNAs) and microRNAs (miRNAs) that are currently the main interest of RNAi therapy, and the recently appreciated single stranded RNAs (ssRNAs) called piwi-interacting RNAs (piRNAs) [40,41].

Cellular Dicer RNAase III endonuclease-mediated maturation of 30 to 100 bp dsRNA are either of endogenic (gene encoded) or exogenic origins and result in the production of ∼21–22 bp long dsRNAs with 3′ two-nucleotide overhangs that are termed siRNA [36,42,43,44]. Following siRNA association with the Argonaute 2 (Ago2) RNA-induced silencing complexes (RISC), the “passenger” siRNA strand (sense strand) is released, whereas the “guide” siRNA strand (antisense strand) remains in the RISC [42,43] to facilitate mRNA target binding and cleavage by Ago2 [43]. Perfect or near-perfect match of the “guide” strand with its mRNA targets leads to mRNA degradation and consequently gene expression silencing [45,46].

RISC complexes can also be occupied by miRNAs, another group of short endogenous ncRNAs [47]. miRNAs genes are first transcribed by RNA polymerase II into long primary miRNA (pri-miRNA) transcripts that are further processed in nucleus by the double-strand-specific ribonuclease, the Drosha-DGCR8 complex, into the precursor miRNA (pre-miRNA) [48]. In the cytoplasm, pre-miRNA are further modified by the Dicer RNAase III endonuclease into mature 21–23 nucleotide miRNAs that enter RISC [20,48,49,50,51,52,53] to either prevent translation or degrade transcripts of their mRNA targets [49,50,51,52,53,54,55,56,57,58,59]. In contrast to siRNA, imperfect complementarity between the nucleotide numbers 2 and 8 of the miRNA strand (the seed sequence) and its mRNA target sequence (located usually within the 3′-untranslated region (3′-UTR) of the mRNAs) is sufficient to obtain functional gene expression silencing [24,25,60,61]. Furthermore, it has been seen occasionally that imperfect complementarity between the siRNA “guide” strand and target mRNA can also result in target translational repression [62].

Dicer-independent mechanisms can produce other small, single-stranded endogenous RNAs from long single-stranded precursors named piRNAs [40]. However, despite the fact that piRNAs recognize their mRNA targets through base pairing, gene silencing does not involve RISC, but rather is mediated by the endonuclease activity of the PIWI proteins [63,64,65,66]. Unfortunately, current understanding of the role these ncRNAs is very limited, especially in somatic cells [40,64,65,67,68,69,70,71,72,73,74,75,76,77,78,79,80] and therefore the potential involvement of piRNAs in RNAi therapeutic approaches remains unclear.

Despite their undisputable therapeutic potential, the delivery pipelines for RNAi drug development and formulation currently struggle with numerous hurdles that impede their clinical use. These hurdles include off-target effects, nonspecific toxicities which may be associated with immunogenic reactions to the dsRNA, immunogenic and non-immunogenic toxicity of excipients, and the unintended activity of ncRNAs or unexpected activity in non-target tissues [32]. Furthermore, the final formulations often have poor metabolic stability, dose limitations, deficient delivery systems, and insufficient therapeutic efficacy [14,32,46,81,82,83]. One of the mainstream solutions to those problems is development of chemical modifications of the RNAi drug candidates’ sequences. These changes inhibit their degradation by endogenous and exogenous nucleases [84,85], enhance guide strand selection and delivery [32,46,86], and can attenuate immune responses. These developments thus improve RNAi drug pharmacokinetics, potency and safety [32,46,86].

Despite the fact that some of the chemical modifications facilitate RNAi drug discharge [32,46,87], however, their efficient and targeted delivery remains one of the major challenges [88]. The native nucleic acid molecules are rapidly degraded in biological fluids. Furthermore, their hydrophilic nature, size (~14–15 kDa) and negative charge inhibits their ability to enter the cell easily through the plasma membrane. The systemic administration of these molecules, therefore, often interferes with their accumulation in target tissues [14,87,89]. A dedicated carrier system needs to prevent them from degradation in the circulation and from their clearance by the mononuclear phagocytic system. The delivery system should also be capable of ensuring that the RNAi drug is delivered into to cytosol and escape endosomal/lysosomal degradation [14,87,89]. Since delivery systems based on viral-derived vectors raises serious safety concerns for patients [90,91,92,93,94,95,96,97,98], chemical excipients that are polymer-based and lipid-based have become the leading strategy for delivering RNAi drugs [32,34,46,81,99,100,101]. Although several approaches have been used in lipid-based systems [88,102,103,104,105], the lipid nanoparticle system (LNPs) is currently the leading technology [106]. LNPs have been shown to be very efficient in hepatocyte transfection due to their liver accumulation and interaction with ApoE [107]. LNPs have also been used in patisiran formulation [16,17]. The polymer-based delivery system utilizes cationic polymers that form electrostatic polyplexes with the negatively charged RNA, and this allows for efficient delivery of RNAi drugs into cells [108,109,110,111,112,113,114,115,116,117,118,119,120,121,122,123]. However, the main limitation of the polymer-based system is their toxicity that results from their high charge density [46,124]. Therefore, lipoplexes consisting of both polymers and lipids are currently being developed to combine both systems delivery advantages [120,125,126,127]. Another potential problems is that, during systemic RNAi drug delivery, their accumulation in non-targeted tissues that may result in toxic effects and this has become major bottleneck for RNAi therapies [128]. Given these potential concerns, the search for efficient, specific, and safe delivery systems continues [32,83,129,130,131,132,133,134].

Although numerous strategies have been undertaken to enhance RNAi drug design, targeted delivery efficiency, and improved bioavailability [41], the risk of off-targets remains a critical challenge. Although siRNA and miRNA design algorithms screen the human genome to avoid candidates with partial homology to other transcripts [135,136,137], these programs usually ignore the SNP-related genetic diversities. The possibility of the individual sequence differences (related to SNPs and the presence of mosaicism) can lead to lowering RNAi drug efficacy (loss or inefficient target binding) or, even worse, the recognition of non-intended sequences and the potential for adverse effects.

## 3. SNPs

SNPs are recognized as the most common type of genetic variation that can be found in the human genome, with a distribution frequency >1% in population [138,139]. They can be located in different regions that include the promoters, exons, introns, and also the 5′- and 3′- UTRs [140]. In this last case, SNPs can be problematic for RNAi therapy as they affect miRNA binding and may alter miRNA-mediated repression of translation [24]. For example, the Gly463Ala polymorphism located in the promoter region of the myeloperoxidase (MPO) gene has been found to be associated with a lower risk of tobacco-induced lung cancers [141,142]. Furthermore, genetic mosaicism that results in cellular genetic diversifications within individual tissues is often associated with carcinogenesis [143]. 

SNPs refer to changes in a single nucleotides located in specific locations in the genome which occur due to base substitution, deletion, or insertion [144,145]. This can result in the production of an altered protein and therefore cause several functional implications, especially if they apply to coding regions (cSNPs) or regulatory regions of genes. The majority of SNPs, however, occur in non-coding regions and they can be found in non-coding RNAs, introns, or in 5′ and 3′ untranslated regions (UTRs) [146,147]. Even if these changes are not responsible for the production of a modified protein associated with particular disease, they can affect RNA-protein interactions through RNA secondary structural changes [148,149,150]. Furthermore, it has been demonstrated that specific SNPs can affect the secondary structures of mRNA [26,151], and this possibility needs to be considered for RNAi therapy development.

SNPs can also affect the genetic susceptibility to cancer and a large number of genes connected with cancer contain SNPs [140]. Despite the fact that one of the most characteristic features of cancer cells is their genetic instability, mostly observed at the chromosome level, the instability is also apparent at the nucleotide level [152]. Moreover, SNPs associated with cancer can affect susceptibility, outcomes and responses to pharmacological treatments [138]. 

The G to A transition at position -463 of the proximal promoter leads to reduced mRNA expression of MPO. A pooled analysis of data from 10 studies (3688 cases) has indicated a protective role of this polymorphism among smokers [153]. Interestingly, the same SNP is associated with a higher risk of gastric cancer connected with *Helicobacter pylori* infection since MPO plays a critical role in host defense against bacterial pathogens. Allele A carriers infected with *H. pylori* are characterized by higher bacterial load, more severe inflammation and neutrophil infiltration, and this leads to glandular damage and atrophy [154]. This emphasizes that another challenge for RNAi therapeutics design will be to distinguish between genes for silencing and gene-related SNPs as well [155].

Several molecular mechanisms concerning region-based and cancer-related SNPs should be highlighted [140]. For instance, if SNPs occur in the promoter region (e.g., TATA box), they will inhibit promoter activity and decrease transcription of the gene. One example is a point mutation in putative TATA box of 17 beta-hydroxysteroid dehydrogenase 2 (*EDH17B2*) gene, which has been suggested as a candidate for the familial breast cancer gene together with the breast cancer type 1 susceptibility protein (*BRCA1*) gene. The analysis revealed that this mutation decreases the activity of the *EDH17B2* in vitro, suggesting that the A nucleotide position is crucial for transcription [156]. On the other hand, SNPs in CpG islands may alter methylation, affect adjacent nonpolymorphic CpG, and the binding of transcription factors. In the case of *BRCA1*, it was found that its downregulation is associated with the methylation status of transcription factors such as specificity protein 1 (SP1) and CCCTC-binding factor (CTCF), which resulted from mutations in their promoter regions [157,158].

In the case of exons, non-synonymous and synonymous cSNPs can occur. The first case results in amino acid substitution because of changes in the first two bases of a codon. This can lead to functional and structural modifications of the translated proteins and disturbances in the cell signaling pathways [140]. For example, cSNPs in epidermal growth factor receptor (EGFR) such as Leu834Arg can lead to the formation of protein dimers and increases cell proliferation [159,160]. On the other hand, mutations such as Thr790Met can affect binding to the EGFR tyrosine kinase domain, which is targeted by tyrosine kinase inhibitors and are essential for the effectiveness of inhibition therapy [161,162]. 

The synonymous cSNPs do not result in the change of amino acid sequence because these nucleotide changes usually occur in the third base of a codon. This often leads to the conclusion that a lack of protein sequence alteration will not have any functional consequences. It has been demonstrated, however, that synonymous cSNPs can affect the expression of neighboring genes, and mRNA splicing, stability, structure, as well as protein function and folding [140,163,164]. For example, these types of mutations in the *KRAS* oncogene were proven to be crucial for protein and mRNA expression and can lead to amplification and overexpression of this gene. As a result of this cSNP, the cell proliferation and metastasis were enhanced, and the resistance to targeted therapeutics was increased [165,166,167,168,169].

SNPs in introns can also regulate protein synthesis as they can affect modulation of mRNA splicing activity and cause changes in the splice donor sites and therefore lead to production of splice variants [140,170,171,172]. Splice site-disrupting SNPs, in rare cases, can lead to the decreased function of a gene [172]. An example of this is the *OAS1* gene (antiviral enzyme 2,5-oligoadenylate synthetase). This SNP disrupts *OAS1* activity and increases the susceptibility of patients to viral infections [172,173,174].

Furthermore, SNPs can also affect functionality of long non-coding RNAs (lncRNAs), another group of regulatory RNAs that participate in chromatin modifications, transcription, and post-transcriptional processing. Notably, to date, 495,729 SNPs have been identified in human lncRNAs transcripts [175], with many of these mutations shown to affect lncRNA structure [176] or their interactions with miRNA [175,177,178]. The SNP-related impairment of lncRNA function is sometimes associated with human diseases including carcinogenesis and responses to chemotherapy [179,180,181,182]. Taken together, it is evident that the cancer-related SNPs can provide important and very selective targets for the development of personalized RNAi therapies.

SNP-directed RNAi drugs, where SNPs have been identified to be causal in a number of diseases including cancers, remain a very promising option [138,183,184,185,186,187,188]. Indeed, *siG12D LODER* is a formulation that is based on a mixture of five siRNAs that target the G12D mutation of KRAS proto-oncogenic GTPase [27,189]. Upon intratumoral injection, *siG12D* is released locally, thereby preventing translation of KRAS proteins and potentially inhibiting growth of tumor cells that normally overexpress KRAS [189]. KRAS, a member of the small GTPase superfamily, is mutated in over 90% of human pancreatic ductal adenocarcinomas (PDAC) and is associated with tumor cell proliferation and reduced patient survival. Despite the fact that KRAS protein mutations are frequently observed in cancers with the glycine 12 mutation, the computational analyses from the Catalogue of Somatic Mutations in cancer revealed nonrandom frequencies of changes of other amino acids as well at this position [190]. KRAS mutations occur overall with a distribution of G12D (42%), 214 G12V (28%), G12C (14%) [190], and G12R (13%) in PDACs [139]. Nevertheless, *siG12D LODER* is currently in phase II clinical trials and provides an elegant example of a drug design strategy that could be applied to cancer-associated SNPs. 

Furthermore, the p53 tumor suppressor (*TP53*) gene has over 200 identified SNPs, and this gene is mutated in in a number of tumor types [191]—although, to date, no population study of sufficient size has reported a correlation between *TP53* SNPs and an altered cancer risk. What they do report is that the polymorphisms that alter p53 function may affect cancer development [191,192]. Notably, the common *TP5*3 codon 72 polymorphism that results in two variants of this protein (Arg72 or Pro72) has been reported to affect the p53 interaction with-NFκB family members, and thus is related to inflammatory responses and carcinogenesis [193,194]. These findings suggest that *TP53* SNPs and mutations may be a potential target for RNAi therapy that selectively eliminates these modified mRNAs that have a dominant-negative effect over the wild type allele or provides survival advantage to the cancer cells. Recent studies have shown that the mutant–p53-specific siRNAs (MupSi) are specific in silencing the expression of the intended mutants without affecting wild-type p53 and effective in inducing cancer cell death [195]. However, applying a similar strategy for *TP53* SNPs will require a better understanding of the underlying molecular mechanisms. 

The lack of consensus in the findings of the role of SNPs in disease, however, is in part influenced by the differences in the study designs and analyses, and the apparent poor understanding of the molecular mechanisms by which particular SNPs contribute to the disease phenotype.

The unanticipated adverse effects of SNPs remain a serious challenge for RNAi drug developers. The mismatches between the siRNAs and their target mRNA can result in partial or total cleavage inhibition by RISC [183,196,197], and this limits their therapeutic potential. Upregulation of vascular endothelial growth factor (VEGF) by oncogenes and hypoxia is considered a key regulator of angiogenesis and is essential for tumor development and growth [198,199,200]. Hence, downregulation of VEGF protein levels has become a goal of novel RNAi therapies, as shown in the recently developed ALL-VSP formulation of systematically-derived siRNA targeting VEGF and KSP (Kinesin spindle protein) [201]. SNPs associated with VEGF are relatively common, however, and are often found to be related to clinical pathology, mortality, and recurrence of cancers [202,203,204,205,206,207]. As already mentioned, the cancer-associated SNPs in VEGF could be potential siRNA targets, although ignoring the possibility of their occurrence in siRNA design would limit siRNA drug efficacy.

SNPs can also introduce novel and unpredicted mRNA targets for both siRNA- and miRNA-based formulations, and this can lead to unique and patient-specific side effects. Furthermore, the genetic diversity of tumor and normal tissues that are related to both SNPs and mosaicism can also lead to nonspecific binding of RNAi drugs to mRNAs in some patients and to “off-target effects in off-target tissues.” Although partial complementarity between siRNA and its mRNA target sequence is usually nonfunctional, in some cases, it can impair translation and lead to protein level reductions [62]. Hence, SNPs can potentially contribute to non-specific target translational repression by siRNA and thus promote adverse effects.

That being said, siRNAs are usually carefully designed to obtain specificity against only one mRNA target and this makes ncRNAs the leading branch of RNAi therapies. Notably, however, siRNA-based therapies are mainly limited to the elimination of target proteins. Promoter sequence specific small activating RNAs that are structurally identical to siRNA, on the other hand, can enhance gene expression [208,209]. Hence, the possibility cannot be excluded that SNPs in promoter regions can create a binding site for siRNAs that were designed specifically for silencing another gene’s expression, and therefore have potential unintended consequences.

Other ncRNAs—miRNA and their analogs, antagomiRs, and agonists for RNA (target protectors/block-miRs)—also provide a therapeutic opportunity for not only silencing gene expression, but also restoring or enhancing protein levels [99]. Indeed, changes in miRNAs expression levels have been shown to be important for cell physiology and development disorders including cardiovascular diseases, diabetes, neurological diseases, and cancer [210,211,212,213]. Moreover, not only oncogenic miRNAs, but also tumor suppressor miRNAs are implicated in tumorigenesis. Downregulation of tumor suppressive miRNAs and upregulation of oncomiRs have been shown to be involved in cancer development [82]. In 2018, approximately 3500 studies about miRNA-based therapeutics were published [81]. Restoration, replacement, or overexpression therapy with miRNA mimics and miRNA reductions with antagomiRs are known as the two miRNA-based strategies for cancer therapies [214,215]. 

The problem of SNPs nonspecifically introducing or impairing mRNA targets, however, is even more serious in the case of miRNA-based RNAi drug candidates since these ncRNAs efficiently repress translation of their target genes based on their imperfect complementarity [216]. Furthermore, a single miRNA can regulate several hundred transcripts functioning at various cellular pathways and networks [40]. miRNAs can also modulate complex signaling networks that are based on diverse autonomous transcription factors [217,218,219], and thus predicting their global impact and adverse effects can be extremely difficult. Therefore, the development of specific miRNA-based therapies can be extremely demanding, and consequently only a limited number of drug candidates have reached clinical trials [15,32,46,83].

SNPs found in the 3’UTR of mRNAs targets can lead to the creation or loss of a binding site for miRNAs and thus alter gene expression [24]. Given the high frequency of SNP occurrences in the human genome along with the partial homology of miRNA target sequences, the occurrence of off-target and adverse effects can be very high. This is especially true for miRNA analogs (mimics) that are delivered at much higher levels than those observed under physiological conditions. miRNA overexpression can lead to saturation of the RISC complexes and recognition of mRNA targets with very little miRNA binding affinity in vivo [46,220]. SNP occurrences can also contribute to this problem by increasing non-physiological target sequence binding. MRX 34, a miRNA drug candidate that was terminated in a phase 1 clinical trial study, is a mimic of miRNA-34. MRX 34 was to be used in patients with advanced solid tumors such as hepatocellular carcinoma, melanoma, small cell lung cancer, triple-negative breast cancer, sarcoma, and bladder, renal, and ovarian cancers. The trial was eventually stopped for safety reasons following multiple immune-related adverse events in the patients. Part of the problem may have been the fact that, under physiological conditions, miR-34 is responsible for downregulation of approximately 30 oncogenes and genes associated with tumor immune evasion [221,222]. Thus, the consequences of the modification of such a wide cellular network affected by miR-34 was extremely hard to predict in patients and diverse and would be potentially dependent upon the patient’s SNPs.

Although poorly understood, the SNPs in human miRNA genes can lead to not only impaired miRNA biogenesis, but also to modification of the miRNA’s target sequence pool of mRNAs [25],86]. The best known experimental miRNA-based drug is Miravirsen used in Hepatitis C treatment [223]. Miravirsen is an antagomiR designed to selectively associate with miR-122 [224]. The major risk factor for hepatocellular carcinoma (HCC) is chronic hepatitis C caused by the hepatitis C virus (HCV). Approximately 70% of total miRNAs pool in hepatocytes is miR-122, making it one of most abundant miRNAs in any tissue. miR-122 plays a pivotal role not only in maintenance of liver homeostasis, but also in hepatitis C virus promotion by protection of the virus from destruction by nucleases [225]. On the other hand, miR-122 has also been reported to be a tumor suppressor against HCC by downregulating target mRNAs involved in cell proliferation, angiogenesis, and apoptosis. In addition to metastasis development, recurrence and poor prognosis of HCC were observed in patients with decreased levels of mir-122 expression [226]. Therefore, HCV infected patients during Miravirsen treatment should be carefully monitored for both liver function and cancer. Bei et al. have shown that common SNPs in the miR-122 gene increase the risk of HCC development [227]. Unfortunately, a similar analysis has not been performed with patients treated with Miravirsen. The SNPs in miR-122 can change the binding affinity between this antagomiR drug and miR-122. Furthermore, the mutations in miRNA seed sequence that modify their biological role could also contribute to cancer development. An example is a common SNP (rs11614913, C→T) in hsa-miR-196a-2 that is associated with an increased risk of breast cancer as well as negative outcomes in non-small cell lung carcinoma (NSCLC) [228,229,230,231].

**Figure 1:**
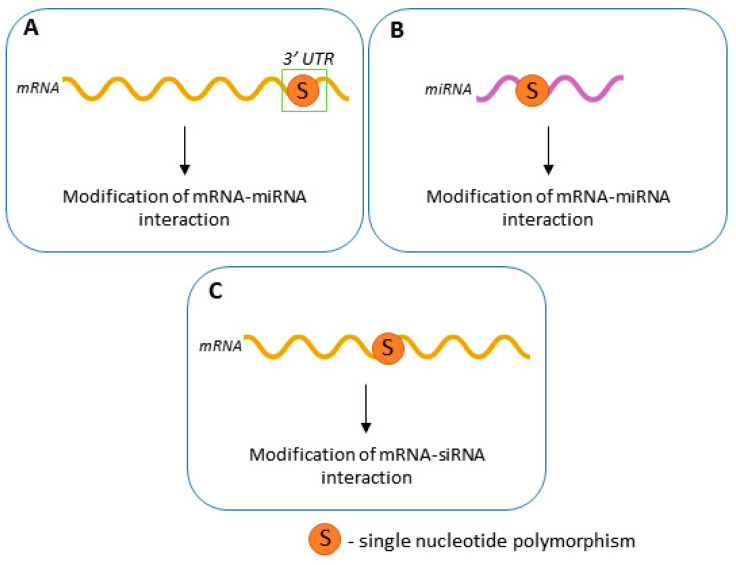
The consequences of SNPs that can affect RNAi therapy (by modifying mRNA-miRNA interactions (**A**,**B**), and by modifying mRNA-siRNA interactions (**C**).

The potential impact of SNPs on RNAi therapy targets has been summarized in Figure 1.

## 4. Conclusions

RNAi drugs provide an opportunity to provide previously untreatable patients with life-changing therapies. Current therapy development focuses on the elaboration of the systemic delivery of RNAi drugs, on the improvement of their pharmacokinetic and pharmacodynamics [32,87,232], and on eliminating the hazards related to their immunogenicity [233,234,235,236,237]. The problems associated with RNAi target sequence-specific recognition still remain partially unresolved [41]. Although the bioinformatic prediction algorithms for designing RNAi drug candidates select them based on a very specific interaction target sequence, these tools utilize consensus genome sequences, and are usually blinded for the occurrence of SNPs [23,24,25,26]. Early predictions of SNP-related adverse effects that included a representative population stratification [238,239,240,241,242] would appear to be more likely to select a safe and reliable RNAi drug candidate. Gaining a better understanding on the role of SNPs in RNAi therapies will dramatically enhance the potential of this technology in the future. Although the generality of SNPs in human population makes both of these approaches extremely challenging, it may soon be more feasible due to the recent affordability of high-throughput genotyping technologies such as deep sequencing and single cell sequencing [243,244,245,246]. Taken together, despite being an obstacle of RNAi therapies, SNPs may also propel their expansion, and therefore SNPs should not be overlooked during RNAi drug development.

## Figures and Tables

**Table 1:** siRNA-based therapeutics under clinical investigation for cancer treatment.

Name	Cancer Type	Target
ALN-VSP	Liver Cancer and metastases	*VEGF* gene, kinesin spindle (KSP) [30,31]
APN401 ^1^	Various solid tumors	CBLB protein [32]
Atu-027	Advanced Solid Tumors/Pancreatic cancer	Protein Kinase N3 gene [30,31]
CALAA-01	Solid Tumors	M2 subunit of ribonucleotide reductase (R2) [31,32]
DCR-MYC	Liver cancer, Multiple myeloma, Non-Hodgkin’s lymphoma, Pancreatic cancer, Solid tumors	*C-Myc* [31]
EphA2	Advanced or Recurrent Solid Tumors	EphA2 [31,32,33]
siG12D LODER	Advanced pancreatic cancer	KRAS G12D [31,32,33]
SPC2996	Chronic Lymphocytic Leukemia	*Bcl-2* gene [30]
TKM 080301	Neuroendocrine tumors (NET) and adrenocortical carcinoma (ACC) tumors	Pololike kinase 1 (PLK1) [33]

^1^ siRNA transfected peripheral blood mononuclear cells.
